# Nationwide Detection and Molecular Characterization of Hepatitis E Virus RNA in Retail Pork Meat in Japan

**DOI:** 10.3390/v18060621

**Published:** 2026-05-29

**Authors:** Masaharu Takahashi, Manri Kawakami, Yukihiro Sato, Tatsunori Nakano, Jun Inoue, Junichi Koyama, Hitoshi Mizuo, Tomoya Koda, Kazumi Yamasaki, Hiroshi Okano, Akio Miyasaka, Shunji Watanabe, Norio Isoda, Tomofumi Takagi, Shinji Fujiwara, Hiroshi Ohnishi, Putu Prathiwi Primadharsini, Shigeo Nagashima, Kazumoto Murata, Hiroaki Okamoto

**Affiliations:** 1 Division of Virology, Department of Infection and Immunity, Jichi Medical University School of Medicine, Shimotsuke 329-0498, Japan; mtaka84@jichi.ac.jp (M.T.); hions.easy@gmail.com (H.O.); thiwik8@jichi.ac.jp (P.P.P.); shigeon@jichi.ac.jp (S.N.); kmurata@jichi.ac.jp (K.M.); 2 Department of Hepatology, Okayama Saiseikai General Hospital, Okayama 700-8511, Japan; 3 Department of Internal Medicine, Kamiichi General Hospital, Nakaniikawa-gun 930-0391, Japan; 4 Department of Internal Medicine, Fujita Health University Nanakuri Memorial Hospital, Tsu 514-1295, Japan; tatsunakano@gmail.com; 5 Division of Gastroenterology, Tohoku University Graduate School of Medicine, Sendai 980-8574, Japan; 6 Department of Internal Medicine, Kin-Ikyo Chuo Hospital, Sapporo 007-8505, Japan; 7 Hokkaido Medical Checkup and Internal Medicine Clinic, Sapporo 060-0032, Japan; 8 Kamikatsu Town Clinic, Katsuura-gun 771-4505, Japan; 9 Clinical Research Center, NHO Nagasaki Medical Center, Omura 856-8562, Japan; 10 Department of Gastroenterology, Suzuka General Hospital, Suzuka 513-8630, Japan; 11 Division of Gastroenterology, Department of Internal Medicine, Iwate Medical University School of Medicine, Shiwa-gun 028-3694, Japan; 12 Division of Gastroenterology, Department of Medicine, Jichi Medical University School of Medicine, Shimotsuke 329-0498, Japan; 13 JCHO Sapporo Hokushin Hospital, Sapporo 004-8618, Japan; 14 Mima Municipal Koyadaira Clinic, Mima 777-0302, Japan

**Keywords:** hepatitis E virus, pork meat, foodborne transmission, zoonosis, molecular epidemiology, nested RT-PCR, Japan

## Abstract

Hepatitis E virus (HEV) is a major zoonotic pathogen, with pigs serving as the principal animal reservoir. While consumption of raw or undercooked pig liver is a well-recognized risk factor, systematic data on HEV contamination in retail pork meat remain limited. In this study, 1546 retail pork samples collected from eight geographic regions across Japan were analyzed for HEV RNA using a validated nested RT-PCR method. Analytical sensitivity was determined using the World Health Organization (WHO) International Standard for HEV RNA, yielding a 95% limit of detection (LOD95) of 134 IU/g (95% CI: 105–193). Overall, 15 samples (1.0%) tested positive for HEV RNA, with no significant regional variation (0.4–1.8%; *p* = 0.8375) or difference between domestically produced (1.0%, 12/1260) and imported pork (1.0%, 3/286; *p* = 0.7478). Viral loads in quantifiable samples (*n* = 11) ranged from 9.3 × 10^2^ to 1.0 × 10^5^ IU/g. Genotyping based on partial ORF2 sequences revealed subtype 3b as predominant (*n* = 7), followed by 3a (*n* = 5), 3f (*n* = 2), and 4c (*n* = 1). Each of the 15 strains showed high nucleotide sequence identity (98.8–100%) to its closest reported Japanese strain(s). Phylogenetic analysis showed that all strains clustered with their closest reported HEV strains, with high bootstrap support, despite genetic diversity within subtypes. These findings demonstrate that retail pork meat in Japan is contaminated with HEV RNA and may represent a potential source of human exposure. However, whether HEV RNA-positive retail pork meat contains infectious virus capable of causing human infection remains to be determined.

## 1. Introduction

Hepatitis E virus (HEV) is a major cause of acute viral hepatitis worldwide and an important zoonotic pathogen. According to the World Health Organization (WHO), an estimated 19.47 million acute infections occur annually, resulting in more than 3450 deaths in 2021 [1]. HEV ranks among the highest-risk viruses for animal-to-human spillover and is recognized as a leading foodborne virus in recent joint assessments by WHO and the Food and Agriculture Organization (FAO) [2,3], underscoring its global significance for both public health and food safety.

Although many HEV infections are asymptomatic and self-limiting, severe disease can occur in vulnerable populations. Pregnant women and individuals with pre-existing liver disease are at risk of acute liver failure, while immunocompromised patients, such as solid organ transplant recipients, may develop chronic hepatitis and cirrhosis [4,5,6,7,8]. In addition, extrahepatic manifestations—including neurological, renal, and hematological disorders—are increasingly recognized [9,10].

Taxonomically, the family *Hepeviridae* comprises two subfamilies and five genera, with *Paslahepevirus balayani* (formerly *Orthohepevirus A*) encompassing all eight human-infecting or potentially human-infecting genotypes (1–8) [11,12]. HEV exists as quasi-enveloped particles in the bloodstream and non-enveloped particles in feces [13,14,15]. Its approximately 7.2 kb single-stranded, positive-sense RNA genome encodes three major open reading frames (ORF1, ORF2, and ORF3), responsible for viral replication, capsid formation, and particle egress and host interaction, respectively [16,17,18]. Genotyping and subtyping are essential for molecular epidemiology and source attribution [19].

The epidemiology of HEV is geographically heterogeneous. In endemic regions of Asia and Africa, waterborne outbreaks caused by genotypes 1 and 2 (HEV-1 and HEV-2) predominate, whereas in industrialized countries, including Japan, infections are mainly sporadic, zoonotic and of genotypes 3 and 4 (HEV-3 and HEV-4) [20,21]. Domestic pigs and wild boars serve as the principal reservoirs, and molecular studies have demonstrated close genetic relatedness between strains of HEV-3 and HEV-4 detected in these animals and those infecting humans [22,23,24,25]. HEV-3 comprises at least 14 subtypes (3a–3m and 3ra), and HEV-4 at least nine subtypes (4a–4i), reflecting substantial genetic diversity [19]. In addition to HEV-1 to HEV-4, other genotypes including HEV-5 to HEV-8 have also been identified in animal hosts and are considered to possess zoonotic potential. Notably, HEV-5 and HEV-6 have been detected in wild boars in Japan [26,27].

Transmission of HEV-3/HEV-4 to humans is primarily associated with the consumption of undercooked animal products, particularly pork [22,28]. Notably, a proportion of pigs entering the food chain remain viremic at slaughter, providing a potential source of contamination of edible tissues [29]. Additional transmission routes include blood transfusion, organ transplantation, occupational exposure, and the consumption of contaminated seafood and produce [30,31,32,33,34,35].

In Japan, HEV infection has been increasingly recognized since the late 1990s, with most clinical cases attributable to HEV-3, occasional cases caused by HEV-4, and rare imported infections with HEV-1 [36,37,38]. Molecular epidemiological studies have demonstrated widespread circulation of HEV-3 and HEV-4 in domestic pigs and wild boars across multiple regions, supporting zoonotic transmission [24,39,40,41]. Multiple subtypes of HEV-3 (including 3a, 3b, 3e, 3f, and 3k) and HEV-4 (including 4c, 4g, and 4i) have been identified in both animal reservoirs and human cases, further supporting foodborne transmission in Japan, are circulating and associated with zoonotic food-borne transmission in Japan [41,42,43,44,45]. However, in a substantial proportion of clinically apparent hepatitis E cases, as well as in most subclinical infections detected by nucleic acid amplification testing (NAT) among blood donors, the source of infection remains unidentified [37,38,46].

Previous studies in Japan have primarily focused on HEV contamination in pig liver. HEV RNA was first detected in retail pig livers by Yazaki et al. [24], with subsequent studies reporting positivity rates of up to 4.9% [47]. Consumption of raw or undercooked pig liver has been clearly associated with sporadic hepatitis E cases [24,47], and zoonotic transmission through wild boar and deer meat has also been documented [25,48].

Despite the recognized risk associated with pig liver, systematic data on HEV contamination in retail pork meat, particularly skeletal muscle, remain limited. This represents a critical gap, given that pork is widely and frequently consumed in Japan. The lack of data on HEV contamination in pork meat may contribute to the large proportion of infections with unidentified sources, including those detected in asymptomatic blood donors [37,38,46]. Therefore, comprehensive surveillance of HEV in retail pork meat is essential to improve risk assessment and to better understand foodborne transmission pathways. HEV RNA was analyzed using a validated nested reverse transcription (RT)–polymerase chain reaction (PCR) method with defined analytical sensitivity.

In this context, the present study aimed to determine the prevalence and molecular characteristics of HEV RNA in commercially available pork meat across Japan. Specifically, we sought to clarify whether pork meat represents a previously underrecognized source of HEV exposure and to provide evidence relevant to food safety and public health risk assessment.

## 2. Materials and Methods

### 2.1. Sampling

**Figure 1:**
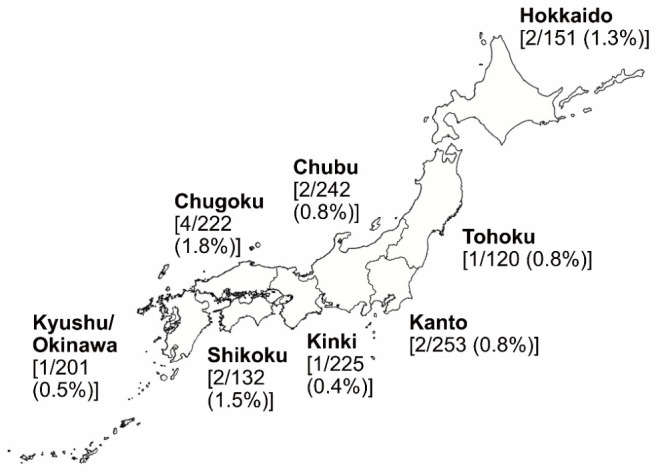
Geographic distribution of retail pork samples collected from supermarkets across eight regions of Japan. The number of HEV RNA–positive samples relative to the total number tested, with percentages, is shown for each region.

From 22 November 2024 to 2 December 2025, a total of 1546 fresh retail pork meat samples were collected from 606 supermarkets located in eight geographic districts in Japan (Hokkaido, Tohoku, Kanto, Chubu, Kinki, Chugoku, Shikoku, and Kyushu/Okinawa) (Figure 1). The number of samples collected per district ranged from 120 to 253. The pork samples consisted of various retail cuts, including loin, pork belly, shoulder loin, sliced/trimmed pork, and pork leg, rather than whole carcass purchases.

For each sample, 10 g of pork was excised from the purchased meat, placed in a plastic bag, and stored at −20 °C. The samples were subsequently transported to our laboratory in frozen batches of ten. Upon arrival, the pork samples were stored at −80 °C until analysis.

To minimize the possibility of obtaining samples originating from the same pig, several measures were implemented. These included purchasing samples from different retail stores whenever possible or, when purchasing from the same supermarket, maintaining an interval of at least one month between purchases or purchasing pork from different production areas. However, potential clustering due to shared supply chains or common processing facilities cannot be fully excluded and may influence prevalence estimates. The number of samples obtained per supermarket ranged from 1 to 25, with an average of 2.6 samples per supermarket.

### 2.2. Homogenization and Nucleic Acid Extraction

During sample preparation, fatty portions were avoided whenever possible, and 100 mg of lean meat was excised from 10 g of pork. The remaining pork was subdivided into 100 mg portions and stored at −80 °C in 2.0 mL Eppendorf tubes until use. The excised sample was finely minced on a disposable plastic dish (Sterile NE Schale; EIKEN Chemical Co., Ltd., Tochigi, Japan) using a surgical blade (Feather Safety Razor Co., Ltd., Osaka, Japan) in the presence of 500 μL of TRIzol reagent (Thermo Fisher Scientific Inc., Waltham, MA, USA).

The minced tissue was transferred to a 1.5 mL tube and homogenized using a BioMasher II (Nippi Incorporated, Tokyo, Japan), followed by the addition of an additional 500 μL of TRIzol reagent. Total RNA was extracted from the homogenate according to the manufacturer’s instructions and dissolved in 50 μL of distilled water.

The RNA solution was further purified using a High Pure Viral RNA Kit (NIPPON Genetics Co., Ltd., Tokyo, Japan). RNA in the eluate was precipitated with ethanol and subsequently dissolved in 6 μL of distilled water containing a ribonuclease inhibitor (RNasin Plus; Promega K.K., Tokyo, Japan). The purified RNA was then subjected to qualitative or quantitative detection of HEV RNA as described below.

### 2.3. Qualitative and Quantitative Detection of HEV RNA

RT–PCR was used to detect HEV RNA. Extracted RNAs (6 μL solution derived from 100 mg meat) were reverse-transcribed with SuperScript IV reverse transcriptase (Thermo Fisher Scientific, Inc.), followed by nested PCR (ORF2/3-143 PCR) using TaKaRa Ex Taq (TaKaRa Bio Inc., Kusatsu, Japan) and 17-mer or 20-mer degenerate primers (HE361, sense primer for 1st round PCR; HE1114, antisense primer for cDNA synthesis and 1st round PCR; HE830, sense primer for 2nd round PCR; and HE1112, antisense primer for 2nd round PCR, respectively), broadly targeting the well-conserved ORF2/3 overlapping region, according to a previously described method [49], with a modified primer derived from HEV sequences that is well-conserved across all eight genotypes (HEV-1 to HEV-8). The antisense primer for 2nd round PCR (HE1113) was replaced with HE1112 (5′-CGCTGGGHYWRRTCDCGCCA-3′ [H = A/T/C, Y = T/C, W = A/T, R = A/G, and D = G/A/T]) in order to make amplification products as long as possible. The first and second rounds of PCR yielded amplification products of 176- and 143-base pairs (bp), respectively. The ORF2/3-143 PCR assay can detect all eight genotypes of HEV strains, as verified in silico using 53 representative sequences from HEV-1 to HEV-8 [19].

For HEV genotyping and confirmation of the presence of HEV RNA detectable by the ORF2/3-143 PCR assay, an additional RT-PCR assay employing primers targeting the ORF2 region (ORF2-457 PCR) [34] was conducted on ORF2/3-143 PCR-positive samples with slight modifications with cDNA synthesis using SuperScript IV reverse transcriptase (Thermo Fisher Scientific, Inc.) and PCR amplifications using TaKaRa Ex Taq (TaKaRa Bio Inc.). The initial PCR amplification product measured 506 bp, and the second-round amplification product was 457 bp in size. The specificity of the RT-PCR assays was validated via a sequence analysis, as described below. The sensitivity of the original ORF2-457 PCR assay has been evaluated as described previously [50].

For the quantification of HEV RNA, real-time RT-PCR (RT-qPCR) was performed using a previously described method [51] with a slight modification. In brief, as a standard, the World Health Organization (WHO) International Standard for HEV RNA provided by the Paul-Ehrlich Institute (PEI code 6329/10) was used in place of a culture supernatant containing a known amount of HEV progeny (1.2 × 10^7^ copies/mL) [52]. The lyophilized standard was reconstituted according to the manufacturer’s instructions and serially diluted in HEV RNA–negative human serum to generate a range of 10^2^ international units (IU)/mL to 10^5^ IU/mL. Total RNA extracted from 100 mg of meat samples was subjected to RT-qPCR using the QuantiTect Probe RT-PCR Kit (Qiagen, K.K., Tokyo, Japan) on a LightCycler apparatus (Roche Diagnostics K.K., Tokyo, Japan). The thermal cycler conditions included incubation at 50 °C for 20 min and initial denaturation at 95 °C for 15 min, followed by 45 cycles of denaturation at 95 °C for 1 s and annealing/extension at 60 °C for 60 s. For pork meat samples, results were expressed in IU per gram (IU/g), calibrated against the WHO International Standard. For clarity, concentrations derived from liquid standards are expressed in IU/mL, whereas those from pork samples are expressed in IU/g. The reproducibility of the quantitative assay was assessed by testing each sample in duplicate, and the mean value was used for analyses.

### 2.4. Determination of Limit of Detection by RT-PCR

The 95% limit of detection (LOD95) for HEV in pork meat by the ORF2/3-143 PCR assay was determined using the WHO International Standard for HEV RNA (PEI code 6329/10). The standard reconstituted according to the manufacturer’s instructions was serially diluted in HEV RNA–negative human serum to generate a range of low-concentration samples spanning the expected detection limit (250, 500, 1000, and 2000 IU/mL). Ten microliters of each dilution were spiked to HEV RNA–negative meat sample (100 mg) and tested in multiple independent replicates (*n* = 20) using the complete extraction and amplification workflow. For each concentration, the number of positive reactions was recorded and expressed as the proportion of positive results. The relationship between HEV RNA concentration and detection probability was analyzed by probit regression analysis, and the LOD95 was defined as the HEV RNA concentration corresponding to a 95% probability of detection. Results were expressed in IU/g, calibrated against the WHO International Standard.

### 2.5. The Determination and Analysis of Nucleotide Sequences

The amplification products were purified using the FastGene Gel/PCR Extraction Kit (NIPPON Genetics Co., Ltd.). Subsequently, both strands were subjected to direct sequencing. Sequencing was performed by FASMAC Co., Ltd. (Atsugi, Japan).

A sequence analysis was performed using the Genetyx software program (version 22; NIHON SERVER Corp., Tokyo, Japan), while multiple alignments were generated using the MUSCLE (multiple sequence comparison by log-expectation) software program, version 3.5 [53]. Phylogenetic tree was constructed based on the 412-nt ORF2 sequence (nt 5944–6355; accession no. M73218), employing the maximum-likelihood method with the Tamura–Nei model, implemented in the MEGA12 software program (version 12.0.14) [54]. Cluster robustness was assessed by executing 1000 bootstrap replicates, and branches with bootstrap values exceeding 70% were grouped together [54]. For comparisons, the proposed reference sequences for HEV subtypes [19] and reported HEV sequences having the highest nucleotide sequence identity to those obtained in the present study were utilized.

### 2.6. Preparation of Inoculum and Cell Culture

Homogenates of pork meat samples were prepared according to a previously described method [52] with slight modifications. Briefly, pork meat samples (100 mg) were minced with a surgical blade and homogenized using a BioMasher II (Nippi Incorporated) in 1 mL phosphate-buffered saline (PBS; pH 7.5). The homogenates were clarified by centrifugation at 7900× *g* for 10 min at 4 °C using a refrigerated microcentrifuge (Tomy Seiko Co., Ltd., Tokyo, Japan), and the resulting supernatants were collected as 10% (*w*/*v*) homogenates.

PLC/PRF/5 cells (ATCC CRL-8024) were cultured and maintained as previously described [52], with minor modifications. Briefly, HEV-infected cells were maintained at 35.5 °C in growth medium supplemented with 1% dimethyl sulfoxide (DMSO; Fujifilm Wako Pure Chemical Corporation, Osaka, Japan) [26]. Culture supernatants were collected every two days and analyzed for HEV RNA by RT-qPCR as previously described [51].

For inoculation experiments, three pork meat samples with relatively high HEV RNA titers (#217, #370, and #697) were selected. The 10% homogenate supernatants were diluted 1:2 or 1:5 with PBS without calcium and magnesium [PBS(–)] containing 0.2% bovine serum albumin (BSA; Sigma-Aldrich, St. Louis, MO, USA), followed by sequential filtration through 0.45- and 0.22-μm membrane filters (Millex-GV; Millipore Corp., Bedford, MA, USA) before inoculation into PLC/PRF/5 cells.

### 2.7. Statistical Analysis

The LOD95 for HEV RNA in pork samples was estimated by probit analysis using the IBM SPSS Statistics [Version: 31.0.0.0 (117)] (IBM Japan, Tokyo, Japan). Detection outcomes obtained from RT-PCR analysis of serially diluted HEV RNA–spiked samples were fitted to a probit model to determine the RNA concentration detectable in 95% of replicates.

For the comparison of positive rate between different sources of sample, chi-square test and Fisher’s exact test were performed using the IBM SPSS Statistics. *p* values of <0.05 were considered significant. The assumptions underlying the chi-square test and Fisher’s exact test, including expected cell counts, were verified prior to analysis. The confidential intervals (CI) at 95% were calculated using Agresti-Coull method for all provided percentages.

### 2.8. Nucleotide Sequence Accession Numbers

The nucleotide sequences of HEV isolates determined in the present study were deposited in the GenBank/EMBL/DDBJ databases with the following accession numbers: LC920989–LC921003.

## 3. Results

### 3.1. Limit of Detection of HEV RNA in Pork Meat by the ORF2/3-143 PCR Assay

The detection limits of HEV RNA in pork meat using the ORF2/3-143 PCR assay are summarized in Table 1. HEV RNA was detected in all 20 pork meat samples (100 mg each) spiked with 10 μL of the WHO standard diluted to 2000 IU/mL (equivalent to 20 IU per sample). Detection was observed in 18 of 20 samples spiked with 10 μL of a 1000 IU/mL dilution (10 IU), in 9 of 20 samples spiked with 10 μL of a 500 IU/mL dilution (5 IU), and in 4 of 20 samples spiked with 10 μL of a 250 IU/mL dilution (2.5 IU). No HEV RNA was detected in any of the 20 unspiked pork meat samples.

Based on these data, the LOD95, calculated using probit analysis, was estimated to be 134 IU/g.

### 3.2. Prevalence of HEV RNA in Retail Pork Meat Stratified by Geographic Region of Purchase

A total of 1546 pork meat samples were collected from eight geographic regions, with the number of samples per region ranging from 120 to 253 (mean: 193.3). These samples were obtained from 606 supermarkets, with 54 to 103 supermarkets sampled per region (mean: 75.8). The average number of pork meat samples purchased per supermarket was 2.6, ranging from 1.7 to 4.2 across regions (Table 2).

Of the 1546 pork meat samples analyzed, 15 (1.0%) tested positive for HEV RNA. The positivity rate ranged from 0.4% to 1.8% and did not differ significantly among geographic regions (chi-square test, *p* = 0.8375) (Table 2, Figure 1).

### 3.3. Comparison of HEV RNA Prevalence Between Domestic and Imported Retail Pork Meat

Among 1546 pork meat samples analyzed, 1260 were domestically sourced and 286 were imported. The domestic samples were obtained from 570 supermarkets, with an average of 2.2 samples collected per supermarket. In contrast, the imported samples were collected from 157 supermarkets, with an average of 1.8 samples per supermarket (Table 3).

The prevalence of HEV RNA was comparable between domestic and imported pork meat, at 1.0% (12/1260) and 1.0% (3/286), respectively. Statistical analysis using Fisher’s exact test indicated no significant difference between the two groups (*p* = 0.7478) (Table 3).

### 3.4. Molecular and Epidemiological Characteristics of 15 HEV Strains Obtained from Retail Pork Meat

Four pork meat samples were below the limit of quantification; however, the viral loads of the remaining 11 samples ranged from 9.3 × 10^2^ to 1.0 × 10^5^ IU/g (Table 4).

Among the 15 HEV strains identified from HEV-positive retail pork meat samples, seven were classified as subtype 3b, followed by five as 3a, two as 3f, and one as 4c. The names and accession numbers of the 15 HEV strains are shown in Table 4.

Analysis of the 412-nt ORF2 region revealed that all 15 HEV strains were genetically distinct from one another, even within the same subtype. The nucleotide sequence identities ranged from 89.6–96.1% for subtype 3a, 85.4 to 97.6% for subtype 3b, and 88.6% for subtype 3f. All strains identified in this study showed the highest genetic similarity to HEV strains of Japanese origin, previously isolated from hepatitis E patients or HEV-NAT–positive blood donors. Notably, each strain exhibited 98.8–100% nucleotide sequence identity with its closest reported HEV strain(s) (Table 5).

**Figure 2:**
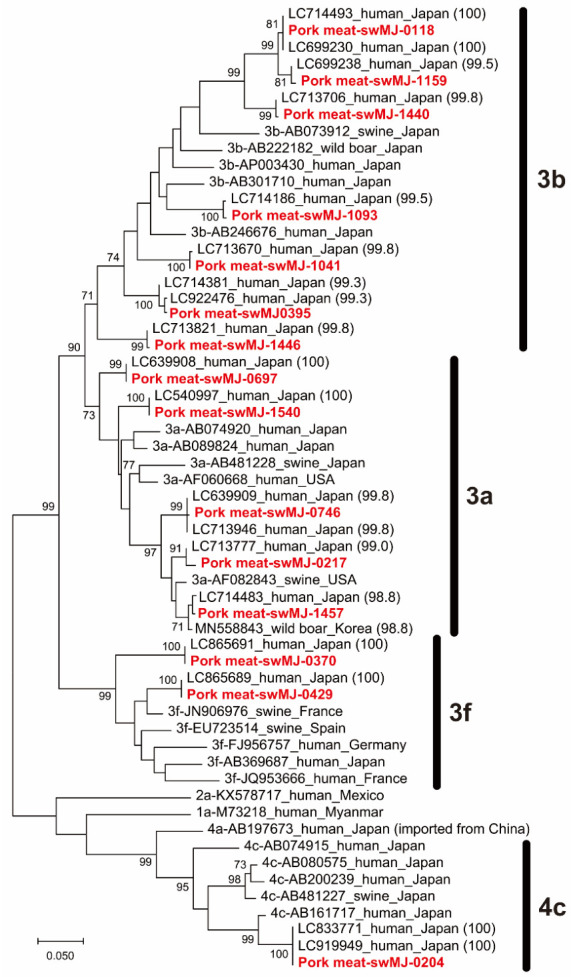
Maximum-likelihood phylogenetic tree based on a 412-nt fragment of the ORF2 region of 15 hepatitis E virus (HEV) strains detected in HEV RNA–positive pork meat samples in this study, together with their closest related reference strains and representative sequences of subtypes 1a, 2a, 3a, 3b, 3f, 4a, and 4c. HEV strains identified in this study (Table 4 and Table 5) are shown with strain names prefixed by “Pork meat” and are highlighted in red and bold. The closest related strains are indicated by their DDBJ/EMBL/GenBank accession numbers, followed by the isolation source, country of origin and nucleotide sequence identity (%) in parentheses. Reference strains [19] are labeled by subtype, accession number, isolation source, and country of origin; one strain each is shown for subtypes 1a, 2a, and 4a, and five strains each for subtypes 3a, 3b, 3f, and 4c. The phylogenetic tree was constructed using the maximum-likelihood method implemented in MEGA12 [54], with optimized tree topology and branch lengths. Bootstrap support values (>70%) based on 1000 replicates are indicated at the nodes. The scale bar represents 0.050 nucleotide substitutions per site.

This finding was further supported by phylogenetic analysis of the 412-nt ORF2 sequences, which demonstrated that all strains clustered with previously reported Japanese HEV strains with high bootstrap support (bootstrap value of 71–100%) (Figure 2).

### 3.5. Comparison of HEV Strains Recovered from Retail Pork Samples in Japan with Reported HEV Strains Focusing on the HEV Subtype

As summarized in Table 4, seven subtype 3b HEV strains isolated from pork samples were all of domestic origin. These strains showed ≥98% nucleotide sequence identity with 1 to 80 previously reported HEV strains, all of which were of Japanese origin (Table 5).

Five subtype 3a strains exhibited ≥ 98% identity with 5 to 21 reported HEV strains. Among these, two strains (swMJ-0217 and swMJ-1457) showed high similarity to HEV strains of non-Japanese origin, including those from the USA, Republic of Korea, and China. Notably, among the two strains derived from imported pork (swMJ-1457 and swMJ-1540), swMJ-1457 showed ≥98% identity with six reported strains, three of which were of non-Japanese origin (Table 5).

Among two subtype 3f strains detected, one (swMJ-0370) was from domestic pork and the other (swMJ-0429) from imported pork. The imported strain (swMJ-0429) showed ≥98% identity with nine reported strains, including eight isolated in European countries (Spain, Germany, and Hungary) (Table 5).

A single subtype 4c strain (swMJ-0204) showed ≥98% identity with 21 reported HEV strains, all of which were of Japanese origin (Table 5).

### 3.6. Inoculation of HEV RNA–Positive Pork Meat Homogenates into Cultured Cells

Filtered homogenates prepared from three HEV RNA–positive pork meat samples with relatively high viral loads (#217, #370, and #697) were inoculated into PLC/PRF/5 cells at 2-fold and 5-fold dilutions. The corresponding inoculum titers were 3.3 × 10^2^ and 1.3 × 10^2^ IU/well for sample #217, 5.7 × 10^2^ and 2.2 × 10^2^ IU/well for sample #370, and 2.2 × 10^3^ and 8.9 × 10^2^ IU/well for sample #697, respectively.

During the 40-day observation period, HEV RNA in culture supernatants remained below the detection limit in all inoculated cultures, and no evidence of viral replication was observed.

### 3.7. Comparison with Reported Studies That Examined the Prevalence of HEV RNA in Pork Meat Obtained from Retail Markets or Slaughterhouses

Among the 14 previously reported studies that examined pork obtained from retail markets or slaughterhouses [55,56,57,58,59,60,61,62,63,64,65,66], six reported no detectable HEV RNA, whereas the remaining eight detected HEV RNA with positivity rates ranging widely from 0.4% to 33.3% (Table 6). Notably, the highest prevalence was reported by Hao et al. [58] in retail pork meat from Yunnan Province, China; however, this finding should be interpreted cautiously because it was based on a relatively small sample size and was markedly higher than most other reports summarized in Table 6. In the present study, HEV RNA was detected in 15 of 1546 pork samples, corresponding to a prevalence of 1.0%. Thus, the positivity rate observed in this study falls within the range reported previously but is markedly lower than the highest values described in the literature and higher than the studies in which no HEV RNA was detected.

## 4. Discussion

The present study demonstrated that HEV RNA was detected in 15 (1.0%) of 1546 retail pork samples collected from eight geographic regions across Japan, with no significant regional variation (0.4–1.8%). Prevalence was identical between domestically produced and imported pork (1.0%). Among the positive samples, viral loads in 11 quantifiable specimens ranged from 9.3 × 10^2^ to 1.0 × 10^5^ IU/g. Genotyping revealed that subtype 3b (*n* = 7) was predominant, followed by 3a (*n* = 5), 3f (*n* = 2), and 4c (*n* = 1). All detected strains were genetically distinct but exhibited high nucleotide sequence identity (98.8–100%) with their closest reported Japanese HEV strains and clustered with those strains with high bootstrap support (71–100%).

Subtype distribution patterns were broadly consistent with known epidemiology. Subtype 3b was exclusively associated with Japanese lineages, supporting its endemic predominance [19,67]. In contrast, subtype 3a showed genetic links to both domestic and international strains, including those from the USA, Republic of Korea, and China, and was also detected in imported pork, supporting global circulation and potential introduction via pork trade [19,67]. Subtype 3f strains were closely related to European lineages, consistent with its predominance in Europe [19,67], while its detection in domestic pork, together with recent reports [43,68], suggests that this subtype may be emerging or becoming established in Japan. The subtype 4c strain was closely related only to Japanese strains, in line with previous observations that it is the most common genotype 4 subtype in Japan [19,46]. In addition to the subtypes identified in the present study, other HEV subtypes including HEV-3e, 3k, 4g, and 4i have also been reported in humans, domestic pigs, and wild boars in Japan [41,42,43,44,45], indicating substantial genetic diversity of zoonotic HEV circulating in the country.

To our knowledge, this is the first nationwide report of HEV RNA prevalence in retail pork meat in Japan. The observed prevalence (1.0%) falls within the range reported in previous studies conducted in other countries, most of which reported 0–12.6% positivity [55,56,57,59,60,61,62,63,64,65,66], with one outlying report from China describing 33.3% positivity (Table 6). Direct comparison remains challenging due to biological and methodological differences.

The relatively low detection rate observed in this study is biologically plausible, as skeletal muscle is not a primary site of HEV replication. Previous studies have consistently shown that HEV RNA is detected more frequently and at markedly higher concentrations in liver tissue, whereas detection in muscle is sporadic and typically at lower levels [24,60,61,62]. This fundamental difference in tissue tropism represents a major determinant of both detection probability and apparent prevalence in pork-based surveys.

In skeletal muscle, HEV RNA is likely unevenly distributed and present primarily as a consequence of systemic infection rather than local replication. Experimental and field studies suggest that viral RNA detected in muscle tissue originates predominantly from residual blood or transient viremia at the time of slaughter, rather than from productive infection of myocytes [29]. Consequently, HEV contamination in pork meat is expected to be focal rather than homogeneous, with viral RNA concentrated in localized regions associated with blood vessels, capillaries, or hemorrhagic residues [69,70,71].

This pronounced spatial heterogeneity has important implications for surveillance studies. Several investigations have shown that small differences in tissue selection, sectioning, or sampling location within the same cut of meat can result in orders-of-magnitude differences in measured HEV RNA levels or even lead to discordant qualitative results [62,72]. As a result, even studies employing similar analytical methods may yield substantially different prevalence estimates, particularly when viral loads hover near the analytical limit of detection. This highlights the importance of standardized sampling protocols to improve comparability across studies.

Moreover, because only a single 100 mg aliquot derived from each 10 g pork sample was analyzed in this study, localized or focal HEV contamination within skeletal muscle may not always have been captured. Therefore, the observed prevalence may underestimate the true frequency of HEV RNA-positive pork meat. Future studies incorporating replicate subsampling, replicate extraction procedures, and repeated molecular testing from the same tissue specimen would help estimate detection probability and improve quantitative interpretation of prevalence data.

Because the proportion of tissue analyzed relative to the total edible pork mass is necessarily small, the probability of HEV RNA detection is influenced not only by analytical sensitivity but also by the spatial distribution of virus within tissue. Accordingly, prevalence estimates obtained using single-aliquot testing should be interpreted as operational detection rates under the defined sampling conditions rather than absolute estimates of carcass-level contamination. Statistical approaches incorporating replicate testing and probabilistic detection modeling may help refine prevalence estimation in future investigations.

In addition, methodological factors play a critical role in shaping reported HEV RNA prevalence in pork meat. Pork matrices are known to contain lipids, proteins, and heme-related compounds that inhibit nucleic acid extraction and polymerase activity, thereby reducing assay sensitivity if not adequately controlled [73,74,75]. Differences in sample homogenization, virus recovery efficiency, and inhibitor removal strategies can therefore strongly influence detection outcomes [76,77]. More extensive homogenization procedures, including homogenization of larger tissue volumes prior to aliquoting, may further reduce potential sampling bias associated with focal viral distribution and could improve representativeness in future surveillance studies.

Furthermore, variability in molecular detection approaches contributes additional inter-study heterogeneity. Comparative studies have demonstrated that detection results can depend on the interaction between matrix type and assay format, with nested RT-PCR and real-time RT-PCR exhibiting differential sensitivity depending on sample composition and viral load range [56]. Even among real-time RT-PCR assays, performance varies according to primer–probe design, target genomic region, and assay optimization for complex food matrices [78,79]. Although RT-qPCR assays are generally considered highly sensitive under optimized conditions, comparative studies have shown that assay performance may vary depending on sample matrix composition, viral load, and amplification design [78]. In complex food matrices containing PCR inhibitors, nested RT-PCR may provide improved sensitivity for low-copy-number targets because the second-round amplification can partially compensate for reduced amplification efficiency in the initial reaction.

Taken together, the wide variation in reported HEV RNA prevalence in pork meat across studies, including the exceptionally high prevalence and viral titers reported by Hao et al. [58] is best interpreted in light of differences in sampling strategies, sample size, tissue selection, and detection methodology, as well as the low and uneven viral distribution expected in skeletal muscle. These factors collectively underscore the need for caution when comparing prevalence estimates between studies and provide an essential framework for interpreting both low detection rates and occasional high viral loads observed in pork meat (Table 6).

Against this general framework, the detection of relatively high HEV RNA titers (up to 10^4^–10^5^ IU/g) in a subset of pork samples in this study warrants focused interpretation. Rather than indicating widespread contamination, these findings are most consistent with sporadic, localized contamination events occurring within otherwise low-prevalence pork meat. Such localized elevation in viral load is plausibly explained by focal retention of virus-rich residual blood in areas with dense vasculature, combined with the intrinsically heterogeneous distribution of HEV RNA in skeletal muscle tissue [29,60,62,69,70,71,72]. In addition, pigs slaughtered during an early or active phase of infection may exhibit transient systemic dissemination, resulting in locally elevated viral RNA levels in peripheral tissues [29,58]. Consistent with this interpretation, the analytical workflow used in this study was validated for pork matrices and exhibited defined sensitivity characteristics, supporting the biological plausibility of the observed quantitative range.

From a risk assessment perspective, such infrequent but high-titer contamination events are noteworthy because they may result in substantially higher potential viral exposure per serving than would be inferred from prevalence estimates alone. Thus, while overall detection rates remain low, the occasional presence of high viral loads in widely consumed pork meat has potential implications for population-level exposure and is therefore relevant to the assessment of foodborne HEV risk.

The estimated annual incidence of approximately 270,000 HEV infections in Japan, based on NAT among blood donors, suggests that a substantial proportion of infections are asymptomatic and remain undetected by routine surveillance [6,46]. This estimate was extrapolated from the nationwide prevalence of HEV NAT-positive blood donors identified through universal donor screening and adjusted using assumptions regarding the duration of detectable viremia in asymptomatic infections [46]. This is consistent with the clinical characteristics of HEV-3, which predominates in industrialized countries and typically causes subclinical or mild disease in immunocompetent individuals [6,20,80,81]. Consumption of raw or undercooked pork liver and game meat is a recognized route of zoonotic HEV transmission [24,25,82,83]; however, these foods are consumed by a relatively small proportion of the population and are unlikely to account for the large number of infections at the population level.

In contrast, pork meat is widely consumed in Japan and may represent an under-recognized route of exposure. Although HEV RNA has been detected in pork products in previous studies, typically at lower levels than in liver [24,47,84,85], Hao et al. [58] reported unusually high titers of 1.2 × 10^5^–1.5 × 10^6^ copies/g in retail pork meat from China. In the present study, viral loads reached up to 10^4^–10^5^ IU/g in some samples, although direct comparison with copy-based values is limited by differences in quantification standards and assay calibration. Given typical portion sizes, ingestion of such meat could result in exposure to substantial amounts of detectable HEV RNA if consumed raw or undercooked. Assuming a typical serving size of approximately 150 g of pork meat, the estimated theoretical exposure dose in the present study would range from approximately 10^5^ to 10^7^ IU in samples with higher viral loads. Although direct conversion between IU-based and copy-based values remains imperfect, these estimated exposure levels may overlap with the experimentally suggested oral infectious dose reported in pig models (approximately 10^5^ genome copies) [86]. This observation may support the biological plausibility that a subset of HEV RNA–positive pork meat samples could represent a potential source of infection if consumed raw or insufficiently cooked. While the detection of HEV RNA does not demonstrate the presence of infectious virus, previous experimental studies have suggested that infectious HEV may not be completely inactivated by inadequate heating, and uneven heat distribution or cross-contamination during food preparation may allow residual virus to persist [87,88].

These findings are consistent with hypothesis that HEV RNA–positive pork meat may contribute to HEV exposure in Japan. Rather than representing a high-risk but infrequent exposure, pork meat may represent a low-risk but potentially frequent source of HEV exposure at the population level. However, this interpretation should be made with caution, given uncertainties regarding infectivity, contamination prevalence, and the minimum infectious dose [89]. Further integrated risk assessments combining virological, epidemiological, and dietary data, as well as molecular linkage analyses, are needed to clarify the contribution of pork meat to HEV transmission.

A key limitation of this study is that infectivity could not be demonstrated despite the detection of HEV RNA in retail pork samples. Although high-titer samples were selected for cell culture, the viral load of the inoculum was reduced below the threshold required to establish infection due to necessary purification steps to remove inhibitors [52]. Consequently, HEV replication was not observed. Therefore, whether HEV RNA–positive pork meat contains infectious virus capable of causing human infection remains to be determined. While muscle tissue is not a primary site of HEV replication, documented cases demonstrating molecular linkage between HEV strains detected in patients and implicated animal meat products, including pork, wild boar, and deer meat [25,48,90], suggest that meat-derived HEV can cause human infection.

Future studies integrating virological data with consumption patterns, dose-response relationship, and molecular epidemiological analyses will be essential to better clarify the public health impact. In addition, standardized methodologies for sampling and detection, as well as improved approaches to assess viral infectivity, are needed to advance risk assessment in this field.

## 5. Conclusions

This study provides the first nationwide evidence that retail pork meat in Japan is contaminated with HEV RNA, with a prevalence of 1.0% and no significant regional differences. The detected strains were genetically diverse yet closely related to domestic lineages, with subtype 3b predominating alongside globally distributed subtypes such as 3a and 3f. Notably, some samples contained relatively high viral loads, indicating that HEV RNA may occasionally be present at substantial levels in pork muscle tissue. Given the widespread consumption of pork, these findings suggest that retail pork meat may represent a previously under-recognized potential source of human HEV exposure. Although the presence of infectious virus could not be demonstrated in this study, the use of a validated detection method supports the reliability of the RNA detection results. These findings highlight the need for standardized detection methods and integrated risk assessments for HEV in pork products. Further studies are needed to determine whether HEV RNA-positive pork meat harbors infectious virus capable of causing human infection and to clarify its role in HEV transmission.

## Figures and Tables

**Table 1:** Limit of detection for HEV RNA using nested RT-PCR (ORF2/3-143nt) in pork meat samples.

HEV RNA (IU/g) ^a^	Number of Replicates Tested	Number of Replicates Detected	% Detected (95% CI)
200	20	20	100 (80.6–100.0)
100	20	18	90 (68.4–98.2)
50	20	9	45 (25.9–65.8)
25	20	4	20 (7.7–42.3)
0	20	0	0 (0.0–19.4)

95% limit of detection (probit analysis) = 134 IU/g (95% CI: 105–193). ^a^ WHO International Standard for HEV RNA, code 6329/10, diluted in normal human serum and spiked in pork meat samples. CI, confidence interval.

**Table 2:** Characteristics of retail pork sampling and prevalence of HEV RNA–positive samples by geographic region in Japan.

Geographic Region of Purchase	Number of Pork Samples Purchased	Number of Supermarkets Surveyed	Number of Pork Samples Purchased per Supermarket (Mean ± SD [Range])	Number of HEV RNA–Positive Samples (% [95% CI])
Hokkaido	151	77	2.0 ± 1.8 (1–12)	2 (1.3 [0.1–5.1])
Tohoku	120	63	1.9 ± 1.6 (1–11)	1 (0.8 [0–5.1])
Kanto	253	92	2.8 ± 2.9 (1–19)	2 (0.8 [0–3.1])
Chubu	242	83	2.9 ± 3.7 (1–23)	2 (0.8 [0–3.2])
Kinki	225	54	4.2 ± 4.1 (1–17)	1 (0.4 [0–2.8])
Chugoku	222	57	3.9 ± 4.6 (1–25)	4 (1.8 [0.6–4.8])
Shikoku	132	77	1.7 ± 1.5 (1–12)	2 (1.5 [0.1–5.8])
Kyushu/ Okinawa	201	103	2.0 ± 1.7 (1–10)	1 (0.5 [0–3.1])
Total	1546	606	2.6 ± 2.9 (1–25)	15 (1.0 [0.6–1.6])

**Table 3:** Prevalence of HEV RNA in retail pork meat according to production origin.

Production Origin	Number of Pork Samples Purchased	Number of Supermarkets Surveyed	Number of Pork Samples Purchased per Supermarket (Mean ± SD [Range])	Number of HEV RNA–Positive Samples (% [95% CI])
Domestic	1260	570	2.2 ± 2.5 (1–25)	12 (1.0 [0.5–1.7])
Imported	286	157	1.8 ± 1.7 (1–14)	3 (1.0 [0.2–3.2])

**Table 4:** Molecular characteristics of HEV detected in retail pork samples collected across Japan.

Sample ID	Geographic Region of Purchase	Production Origin	HEV RNA Concentration (IU/g of Meat)	HEV Subtype	Name of HEV Strain	Accession Number
118	Chubu	Domestic	1.1 × 10^4^	3b	swMJ-0118	LC920989
204	Hokkaido	Domestic	<5.0 × 10^2^ *	4c	swMJ-0204	LC920990
217	Kanto	Domestic	3.4 × 10^4^	3a	swMJ-0217	LC920991
370	Tohoku	Domestic	3.1 × 10^4^	3f	swMJ-0370	LC920992
395	Chugoku	Domestic	<5.0 × 10^2^	3b	swMJ-0395	LC920993
429	Chugoku	Imported	9.3 × 10^2^	3f	swMJ-0429	LC920994
697	Kinki	Domestic	1.0 × 10^5^	3a	swMJ-0697	LC920995
746	Chubu	Domestic	7.4 × 10^3^	3a	swMJ-0746	LC920996
1041	Shikoku	Domestic	2.8 × 10^3^	3b	swMJ-1041	LC920997
1093	Hokkaido	Domestic	1.1 × 10^3^	3b	swMJ-1093	LC920998
1159	Kyushu	Domestic	1.6 × 10^3^	3b	swMJ-1159	LC920999
1440	Chugoku	Domestic	<5.0 × 10^2^	3b	swMJ-1440	LC921000
1446	Chugoku	Domestic	<5.0 × 10^2^	3b	swMJ-1446	LC921001
1457	Shikoku	Imported	1.2 × 10^4^	3a	swMJ-1457	LC921002
1540	Kanto	Imported	6.5 × 10^3^	3a	swMJ-1540	LC921003

* Below the limit of quantification.

**Table 5:** Comparison of HEV strains recovered from retail pork samples in Japan with reported HEV strains.

Name of HEV Strain	Accession Numbers of the Reported HEV Strains with the Highest Nucleotide Sequence Identity (%)	Number of Reported HEV Strains with the Indicated Nucleotide Sequence Identity (%) of:		
		100	99.0–99.9	98.0–98.9
**Subtype 3b**				
swMJ-0118	LC699230, LC714493 (100)	2 (Japan) ^a^	8 (Japan)	70 (Japan)
swMJ-0395	LC714381, LC922476 (99.3)	0	5 (Japan)	6 (Japan)
swMJ-1041	LC713670 (99.8)	0	1 (Japan)	0
swMJ-1093	LC714186 (99.5)	0	4 (Japan)	28 (Japan)
swMJ-1159	LC699238 (99.5)	0	4 (Japan)	64 (Japan)
swMJ-1440	LC713706 (99.8)	0	7 (Japan)	5 (Japan)
swMJ-1446	LC713821 (99.8)	0	2 (Japan)	0
**Subtype 3a**				
swMJ-0217	LC713777 (99.0)	0	1 (Japan)	3 (Japan), 1 (USA: AF466677)
swMJ-0697	LC639908 (100)	1 (Japan)	1 (Japan)	12 (Japan)
swMJ-0746	LC639909, LC713946 (99.8)	0	14 (Japan)	10 (Japan)
swMJ-1457 ^b^	LC714483, MN558843 (98.8)	0	0	3 (Japan), 1 (Republic of Korea: MN558843), 1 (China: KF691585), 1 (USA: AF466677)
swMJ-1540 ^b^	LC540997 (100)	1 (Japan)	8 (Japan)	12 (Japan)
**Subtype 3f**				
swMJ-0370	LC865691 (100)	1 (Japan)	1 (Japan)	0
swMJ-0429 ^b^	LC865689 (100)	1 (Japan)	1 (Spain: PX988439)	5 (Spain: OP793790, PX988425, MZ272554, MZ272562, MZ272610), 1 (Germany: MZ814750), 1 (Hungary: PV877311)
**Subtype 4c**				
swMJ-0204	LC833771, LC919949 (100)	2 (Japan)	6 (Japan)	13 (Japan)

Only reported HEV nucleotide sequences containing ≥ 300-nucleotide overlapping regions within ORF2 were included in the comparison. ^a^ The country of origin of each HEV strain is indicated in parentheses. For strains isolated outside Japan, the corresponding DDBJ/EMBL/GenBank accession number is also provided in addition to the country name. ^b^ Imported.

**Table 6:** Reported prevalence of HEV RNA in pork meat obtained from retail markets and slaughterhouses in various countries.

Country	Location of Sampling (Muscle Type)	Processing of Meat Before RNA Extraction ^a^	HEV RNA Prevalence (%) [RT-PCR]	HEV RNA Titer (Cq Value or Copies/g)	Reference
Canada	Slaughterhouse (Loins)	Homogenization	0/43 (0) [Real-time]	NA	Leblanc et al. [55]
	Retail markets (Pork chops)	Homogenization	0/599 (0) [Nested]	NA	Wilhelm et al. [56]
China	Slaughterhouses/ retail markets	Homogenization	0/158 (0) [Nested]	NA	Geng et al. [57]
	Retail markets	ND	19/57 (33.3) [ND]	1.2 × 10^5^– 1.5 × 10^6^	Hao et al. [58] ^b^
Czech Republic	Slaughterhouse (Lingual muscles)	Homogenization	1/40 (2.5) [Real-time]	ND	Di Bartolo et al. [59]
France	Slaughterhouse (Gluteus medius or semi-membranosus)	One cycle of freeze and thaw at −20 °C	0/1034 (0) [Real-time]	NA	Feurer et al. [60]
Italy	Slaughterhouse (Lingual muscles)	Homogenization	2/33 (6.1) [Real-time]	ND	Di Bartolo et al. [59]
	Slaughterhouse (Diaphragm)	Homogenization	8/585 (1.4) [Real-time]	Below the quantification limit	Chelli et al. [61]
	Slaughterhouse (Diaphragm)	Homogenization	8/236 (3.4) [Nested]	10^2^ (30.4–37.5)	Ferri et al. [62]
Spain	Slaughterhouse (Lingual muscles)	Homogenization	0/39 (0) [Real-time]	NA	Di Bartolo et al. [59]
	Slaughterhouse (Diaphragm)	Homogenization	1/45 (2.2) [Real-time]	36.0	Garcia et al. [63]
Thailand	Fresh markets	Minced into small pieces (~1 mm^3^)	2/559 (0.4) [Semi-nested]	ND	Intharasongkroh et al. [64]
UK	Processing plant (Ventral abdominal muscles)	Homogenization	0/40 (0) [Real-time]	NA	Berto et al. [65]
USA	Grocery stores (Ground pork)	Homogenization	15/119 (12.6) [One-step (40 cycles)]	ND	Harrison et al. [66]
Japan	Supermarkets (Retail pork meat)	Homogenization	15/1546 (1.0) [Nested]	<5.0 × 10^2^– 1.0 × 10^5^	This study

NA, not applicable; ND, not determined or described. ^a^ Methodological differences among studies included variation in tissue sampling procedures, homogenization approaches, extraction workflows, and molecular detection assays, which may influence reported HEV RNA prevalence. ^b^ This study by Hao et al. was a letter reporting a small sample set and unusually high prevalence and titers; therefore, direct comparison with larger surveillance studies should be made cautiously.

## Data Availability

All data are presented in the manuscript. The sequence data presented in this study are available in the DDBJ/EMBL/GenBank databases: LC920989–LC921003.

## Abbreviations

The following abbreviations are used in this manuscript:

HEV	hepatitis E virus
WHO	World Health Organization
FAO	Food and Agriculture Organization
ORF	open reading frame
HEV-1/2/3/4	HEV genotype 1/2/3/4
NAT	nucleic acid amplification testing
RT	reverse transcription
PCR	polymerase chain reaction
bp	base pairs
RT-qPCR	real-time RT-PCR
IU	international units
LOD95	95% limit of detection
CI	confidential intervals
